# Optimizing crop rotations via Parrondo’s paradox for sustainable agriculture

**DOI:** 10.1098/rsos.221401

**Published:** 2023-05-17

**Authors:** Chaitanya S. Gokhale, Nikhil Sharma

**Affiliations:** ^1^ Center for Computational and Theoretical Biology (CCTB), University of Würzburg, Würzburg, Germany; ^2^ Research Group for Theoretical Models of Eco-evolutionary Dynamics, Department of Evolutionary Theory, Max Planck Institute for Evolutionary Biology, August-Thienemann-Straße 2, 24306 Plön, Germany; ^3^ Department of Evolutionary Theory, Max Planck Institute for Evolutionary Biology, August-Thienemann-Straße 2, 24306 Plön, Germany

**Keywords:** crop rotation, Parrondo’s paradox, fluctuating selection, crop yield, soil quality, fertilizers

## Abstract

Crop rotation, a sustainable agricultural technique, has been at humanity’s disposal since time immemorial and is practised globally. Switching between cover crops and cash crops helps avoid the adverse effects of intensive farming. Determining the optimum cash-cover rotation schedule for maximizing yield has been tackled on multiple fronts by agricultural scientists, economists, biologists and computer scientists, to name a few. However, considering the uncertainty due to diseases, pests, droughts, floods and impending effects of climate change is essential when designing rotation strategies. Analysing this time-tested technique of crop rotations with a new lens of Parrondo’s paradox allows us to optimally use the rotation technique in synchrony with uncertainty. While previous approaches are reactive to the diversity of crop types and environmental uncertainties, we make use of the said uncertainties to enhance crop rotation schedules. We calculate optimum switching probabilities in a randomized cropping sequence and suggest optimum deterministic sequences and judicious use of fertilizers. Our methods demonstrate strategies to enhance crop yield and the eventual profit margins for farmers. Conforming to translational biology, we extend Parrondo’s paradox, where two losing situations can be combined eventually into a winning scenario, to agriculture.

## 1. Introduction

‘In an April speech to Congress […] President Biden suggested paying farmers to plant cover crops, which are grown not for harvest but to nurture the soil in between plantings of cash crops’ [1].

In the coming 30 years, the world will be facing a severe dearth of food [2]. Sufficient nourishment will be hard to provide unless crop yields improve [3,4]. Furthermore, farmers have to face the consequences of climate change, resistant pathogens and recurrent diseases. Fixing the dire situation using intensive cropping techniques and blanket use of fertilizers is a short-term solution and not sustainable. For example, the intensive use of inorganic fertilizers can negatively impact the ecosphere, accelerating climate change [5]. While most of the classical ‘old-fashioned’ strategies, such as tilling and slash and burn agriculture, are not scalable and climate-friendly, perhaps some of the strategies can be upgraded for the future. For hundreds of years, farmers have been rotating crops as a sustainable strategy. The approach has been augmented technologically [6,7] and analysed computationally [8] to provide better crop rotation schedules. Crop rotations are akin to fluctuating selection regimes where the soil quality changes with the planted crops, affecting the following yield. An extremely simplified version assumes that cash crops yield profit, whereas cover crops do not. Cover crops can act as carbon sequestration tools, generally enriching the soil with nutrients and preventing leaching into the water table. Rotations and pairing of cash (such as rice, wheat, maize, sugar beet, asparagus, onions) and cover (such as clovers, buckwheat, black oats, radish) is a complex task [9] since although cover crops improve soil health and prepare it for intensive use for the cash crops, only depending on covers is typically an economic dead-end for the farmers [10].

Conservation agriculture, crop rotations, the impact of cover crops on soil quality and the definition of soil quality itself are extremely complex terms [11]. We have abstracted the definitions to their minimum required properties to observe crop rotations from a different perspective. Considering profit-making in terms of game theory, planting cash crops is a winning game while cover crops are not. However, even cash crops are a losing venture in the long run as the soil quality depletes beyond repair. We use this simplicity of logic as a starting point to connect this complex scenario to a well-studied, seemingly unintuitive phenomenon—Parrondo’s paradox [12,13], combining two losing strategies results in a winning strategy. This seemingly paradoxical behaviour exists in other fields as well. Numerous situations exist across the complexity of life where particular traits, strategies, or behaviours can be detrimental to the organism when implemented over long periods. However, by combining the individually harmful strategies in a specific manner, a sustainable method emerges [14,15]. Starting at the genetic scales, [16] showed how an autosomal allele with a lower fitness advantage could persist and even continue to fixation via its epistatic interactions with alleles at other loci. At the population level, persistence in sink environments is possible if offsprings are distributed over different habitats that fluctuate temporally [17]. Switching between two interaction patterns, competitive and cooperative, has the potential to delay ageing in multicellular organisms [18]. Similar dynamics is observed in bacteriphages that optimize switching between lytic and lysogenic phases [19]. In [20], it is shown how environmental sensors with low accuracy can indeed be selected under certain environmental conditions. In a similar vein, Tan & Cheong [21] show how switching between two evolutionary strategies, nomadism and colonialism, that individually lead to extinction, can lead to population persistence and long-term growth. Concerning grasslands, a Parrondo-like effect explains the persistence of rare forb species existing in competition with the dominant grasses in California under fluctuating environmental conditions [22]. In this study, we leverage this theoretically versatile framework of Parrondo’s paradox to devise profitable and sustainable agricultural practices.

Planting cash and cover crops act as games in our model. The farmer is interested in generating a profit that we assume is directly proportional to the yield. Consistently planting cover crops while increasing soil quality is assumed to provide meagre monetary gains that decrease over time [10]. Similarly, sequentially planting only cash crops will deplete the soil quality over time, making the field barren. Numerous approaches have been proposed to increase crop yield by optimizing the rotation patterns between cash and cover crops, both empirically and computationally [6,7,23]. Considering the spatial and temporal variation in the planted crops, market effects of demand and supply trends, pathogen evolution dynamics and weather conditions, these models can be incredibly complicated. However, not all such approaches are agriculturally sustainable as we move forward [24]. Crop rotations have been proposed as a means of tackling multiple issues of modern-day agriculture—being able to control insects, pests and diseases of crops, control weeds, increase crop yields, lower economic risk, reduce toxic substance accumulations in soils, achieve abundant and lasting soil cover, maintain or increase soil organic matter (SOM) content, provide alternating root systems allowing for a stable extraction of nutrients favouring soil equilibrium, improve soil structure and ultimately promote greater biological diversity [25]. Crop rotation, are thus, a sustainable component of conservation agriculture able to increase the SOM and physical soil protection [26,27]. However, a significant obstacle lies in deriving optimum rotation schedules due to external uncertainties.

Visualizing crop rotations through the lens of Parrondo’s paradox allows us to derive the optimum cropping frequency of cash and cover crops for randomized cropping sequences. With fertilizers, we show that we can increase the range of the profitable cropping frequency given the appropriate cash crops. This helps us identify profitable fertilizer–crop combinations. The general result can be tailored to the specific properties of the crops, such as the threshold soil quality required to grow the cash crop and how fast the cover crop can replenish the soil quality. The probabilities of profit making are often complex combinations of social, economic and evolutionary parameters such as the presence and intensity of crop pests like pathogens and weeds [8,28]. For crop systems, these probabiltities can then be estimated to develop a system-specific model. Even for deterministic sequences, we observe a Parrondo-like effect. Our study thus provides a novel take on determining the optimum crop rotations schedules for maximizing the profit for farmers while simultaneously being a sustainable agricultural strategy.

## 2. From soil to profit

**Figure 1.**
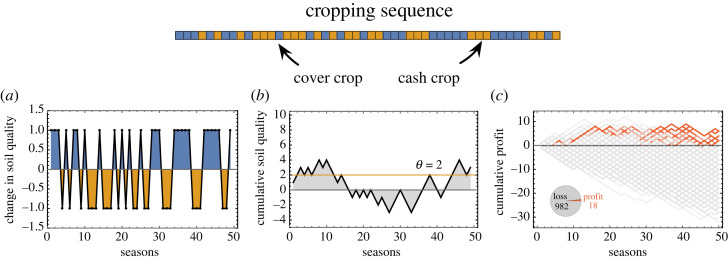
Crop rotations, soil quality and profit margins. Cash and cover crops are highlighted in an exemplar rotation sequence of 50 seasons on top. (*a*) For each season of cash or cover crop, the soil quality decreases or increases by a unit amount. (*b*) The cumulative soil quality over the duration of the sequence is shown. The threshold soil quality required for generating a possibly profitable cash crop is set to *θ* = 2. (*c*) To capture the fickle nature of agricultural outcomes due to various factors, the probabilities of profits possible for the cover crop is set to *p* = 0.2 and for the cash crop in poor soil (soil quality ≤*θ*) is *p*_1_ = 0.5 and *p*_2_ = 0.9 otherwise. The profit is accrued from the end of the first season onwards (hence the lines starting at season = 1). Using these probabilities, we simulate 1000 trajectories. Of the independent runs only a few (18/1000) end up in making a cumulative profit (shown in red, that end up above the 0 cumulative profit line).

The soil quality has a profound effect on the crop yield. Reaping crops for nutritional benefit inherently strips the soil of essential nutrients. Under crop rotations, typically, two types of crops are considered—‘cover’ crops and ‘cash’ crops. Following [8], we define cash crops as those providing a commercialized output, e.g. maize. Cover crops improve the soil quality of the field but provide no direct, substantial cash yield, e.g. clover. Thus, cover crops provide a low yield, if any, but increase the quality of the soil. Alternatively, they can be used as fodder for animals. Cash crops are the main profit-generating crops, such as essential grains like wheat and maize. If the soil quality is above a specific threshold value, then the cash crops can maximally extract the nutrients. We define this threshold soil quality to be *θ*. Thus profits are more probable if cash crops are grown in a field with a good soil quality (soil quality greater than *θ*). A cropping sequence simplifies the use of cash and cover crops where each crop exists for the same unit of time, at the end of which the crop is harvested. This set-up is inspired by a 9-year long field study and tries to capture the essential elements of the sequence [29]. Extended periods of cash or cover crops can be included by consecutive instances of the same type of crop in a sequence. An example cropping sequence between cash and cover crops, the effect on how the soil quality changes with every cropping season and the cumulative effect on the field is shown in figure 1. Profit can be generated (with a certain probability) if the soil quality is higher than the threshold *θ* required by the cash crop (shown by *θ* = 2 in figure 1*b*).

The profit (proportional to the crop yield) depends on the soil quality and other complex external factors, e.g. climate conditions, pathogen coevolution and market volatility [8,30,31]. We have captured these complex interactions by introducing probabilistic outcomes. We denote *p* as the probability of obtaining financial profit from a cover crop (usually minimal). For the cash crops, *p*_1_ and *p*_2_ are the probabilities of obtaining profits when the soil quality value is bad (soil quality less than or equal to *θ*) or suitable (soil quality greater than *θ*), respectively. The decision trees for the cash and cover crops are visualized in the electronic supplementary material, figure A1. Typically, we will have *p*_1_ < 0.5 and *p*_2_ > 0.5. The effect of including the probabilities of profit for a given rotation sequence is shown in figure 1*c*.

So what should be the ideal rotation sequence that maximizes profit? In our previous study [8], the optimum sequence was found by performing an exhaustive search of all possible sequences of a given length. Various computational and empirical approaches have striven to answer this pertinent question [6,7,32,33]. Since the space of possible sequences can be massive, randomized switching provides an excellent analytical handle helping us understand different regions of the parameter space.

## 3. Randomized cropping sequences

We model the effect of different crops on soil quality as a discrete-space discrete-time random walk problem. The soil quality can vary from 0 to a maximum possible value of *K*. We generate a random sequence of cash and cover crops. Electronic supplementary material, figure A1 illustrates this choice based on *γ* between the cash and cover crop decision trees. A cover crop is chosen with probability *γ*, and it is assumed to increase the soil quality by *a* units. Conversely, with probability 1 − *γ*, a cash crop is chosen for the next season, depleting the soil of nutrients and reducing the quality by *b* units at the end of the season (see electronic supplementary material, figure A2). From the Markov chain analysis (electronic supplementary material, §1.2 Discrete Markov chain analysis), the probability of making a profit for the randomized switching is then

**Figure 2.**
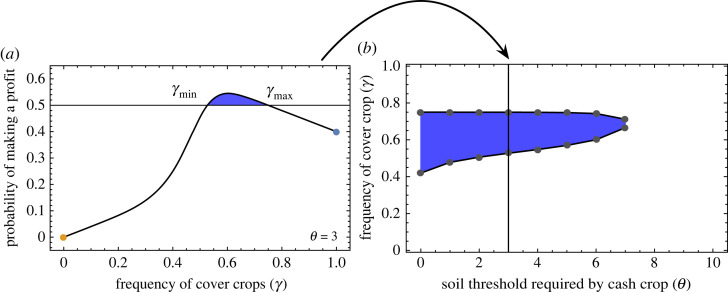
Optimum cover crop frequency and threshold dependence. (*a*) The probability of profiting ($P_{\mathrm{win}}^{R}$) is plotted as a function of the frequency of cover crops (*γ*) used to generate a randomized sequence. We designate a ‘win’ when this probability is more than 50%. The range of the frequency of cover crops where the profit is a ‘win’ is bounded by *γ*_min_ and *γ*_max_. This range is shown for a given threshold value of soil quality *θ* = 3. (*b*) As the threshold soil quality for a cash crop to generate profit increases, the range of *γ*_min_−*γ*_max_ shrinks. Other parameter values are *K* = 10 for the maximum soil quality, and the probability of making a profit on a cover crop to be *p* = 0.4. We assume the probability of making a profit from a cash crop when the soil quality is bad is *p*_1_ = 0 and the under the best conditions is *p*_2_ = 0.8. See electronic supplementary material, figure A1 for further explanation.

**Figure 3.**
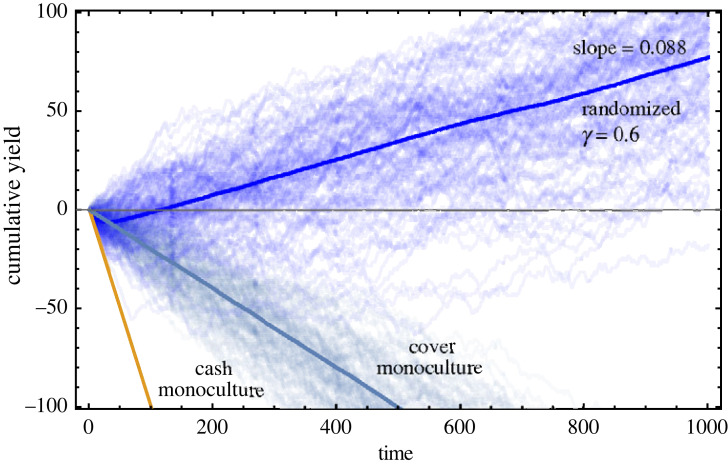
Parrondo effect. We plot the yield for example cropping sequences with different cash–cover proportions (*γ*). The yield is cumulative over the number of seasons (time). The two games—cash monoculture (*γ* = 0) and cover monoculture (*γ* = 1) are shown in orange and cobalt blue, respectively. Mixing the two games (here *γ* = 0.6) leads to the Parrondo effect where the eventual cumulative yield is positive. The parameters used are *θ* = 2, *p* = 0.4, *p*_1_ = 0, *p*_2_ = 0.8 and *K* = 10 with *a* = *b* = 1 (equal jump sizes in the forward and backward direction). Profit is characterized by a unit increase in yield, +1 and a loss by unit decrease, −1. Individual trajectories of the simulations, shown in thin lines, are done with *n* = 1000 (number of seasons per sequence) and *m* = 10 000 (number of cropping sequences). The means of the simulations are shown by solid lines. We find a good agreement between the $P_{\mathrm{win}}^{R}$ computed from the numerical approach using electronic supplementary material, equation A.8 (0.544) and simulations fit using electronic supplementary material, equations A.8, A.9 (0.5372).

3.1 $$P_{\mathrm{win}}^{R}=q_{1}\sum_{i=0}^{\theta }x_{i}^{*}+q_{2}\sum_{i=\theta +1}^{K}x_{i}^{*},$$

where *q*_*i*_ = *γp* + (1 − *γ*)*p*_*i*_, where *i* = 1 or 2 and *x*_i_* is the steady-state probability of soil quality equal to *i* units. When *γ* is 0 or 1, we have cash or cover crops monoculture. For only cash (cover) crop, we have $P_{\mathrm{win}}^{R}=p_{1}$ ($P_{\mathrm{win}}^{R}=p$). It is so because, when only growing cash crops, the field will ultimately become barren. Thus the probability of making a profit becomes *p*_1_ (set to *p*_1_ = 0). On the other hand, with prolonged use of cover crops, although the soil quality reaches the carrying capacity *K*, the probability of winning remains *p* (set to *p* = 0.4). Since both *p* and *p*_1_ are less than half, under monoculture the profits always dwindle. We highlight these probabilities in figure 2*a*, as the extremes of the curve representing the cash monoculture (*p*_1_ = 0—yellow marker) and cover monoculture (*p* = 0.4—purple marker). However, something unintuitive happens for the intermediate values of *γ*. For a given set of parameters, we find that $P_{\mathrm{win}}^{R}>0.5$ for a certain range of *γ*, the blue shaded region of figure 2*a*. That is, we can make a profit by randomly switching between two cropping strategies that individually result in a loss figure 3. This is an effect similar to the famous Parrondo’s paradox, where two individually losing games combine to form a winning game [12]. The choice of values for *p*, *p*_1_ and *p*_2_ follows the intuitive logic as described above (and in electronic supplementary material, figure A1). A parameter sweep within this logical condition is possible if we include an economic aspect to our model. Then we can connect the yield in monetory terms, but such an extension is currently beyond the scope of this study.

We further find that the range of *γ*, the frequency of cover crops, for which $P_{\mathrm{win}}^{R}>0.5$, shrinks with an increase in soil quality threshold *θ*. It can be seen in figure 2*b* that *γ*_min_ increases with *θ* while *γ*_max_ remains almost constant. Thus as the threshold soil quality increases, the optimal sequences are made up of similar frequency of cover crops. The observation can be explained as follows. For all values of *γ*, *q*_1_ < *q*_2_ (except for *γ* = 1 when *q*_1_ = *q*_2_). On increasing *θ*, we increase the magnitude of the first term on the right-hand side of equation (3.1). However, it is the second term that has a substantial contribution to $P_{\mathrm{win}}^{R}$. To compensate for the effect of increasing *θ*, *γ*_min_ also increases, putting more weight in the tail of the soil quality distribution and hence the second term of equation (3.1).

We define a critical value of the soil quality θ*, such that if *θ* ≤ θ*, $P_{\mathrm{win}}^{R}>0.5$ for some values of *γ*. From figure 2*b*, we find that both *γ*_min_ and *γ*_max_ take values close to 0.5 for *θ* ≤ θ*, indicating the need to switch often between the cash and cover crop to make profit. The equal switching regime also agrees with the classical Parrondo’s game. Switching between games is often done to exploit the asymmetry in the winning probabilities of the sub-games. If the cash crop depletes the soil quality at a very high rate, it is more difficult to make a profit. This can be seen from electronic supplementary material, figure A5, where θ* decreases with the increase in *b*/*a*.

Nevertheless, what if the required soil quality threshold *θ*, for a given cash crop, is above θ*? How do we make a profit in that case? The answer can lie in the efficient use of fertilizers. Using suitable fertilizers depending on the crop system, one can increase the θ*.

### 3.1. Smart fertilizer use

**Figure 4.**
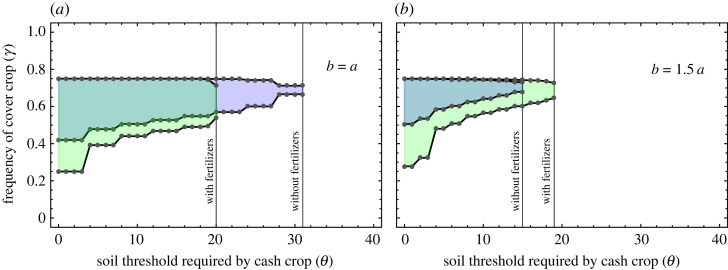
Choosing fertilizers according to crop properties. In panel (*a*), in one season, the cash crop depletes the soil quality with the amount equivalent to that a cover crop recovers in one season. Without fertilizers the threshold value below which some profit is possible, i.e. *P*_win_ > 0.5 is θ* = 31, but the addition of fertilizers reduces this region, limiting to θ* = 20. Conversely, as shown in (*b*), if the cash crops deplete soil quality more than the cover crop can replenish in one season (by *b* = 1.5 *a*), then the addition of fertilizers increases the value of θ* from 15 to 19. Overall the figure conveys the need to be judicious about fertilizer use since θ* can either decrease (see panel (*a*)) or increase (panel (*b*)) depending on the specific choice of cash–cover pairs. Other parameters are *K* = 40, *p* = 0.4, *p*_1_ = 0, *p*_2_ = 0.8.

Continuous exploitation of soil leads to nutrient depletion. Leaving the land fallow for a while or actively planting nutrient enriching cover crops can help recover the soil quality [24,25]. Even crop rotations are typically not enough to maintain stable productivity over several years; interventions to add nutrients are therefore necessary [25]. Thus, fertilizers can be applied to minimize the time the land is left fallow. Often, fertilizers are tailored towards particular cash crops, and an optimal fertilizer–crop combination can lead to enhanced profits. In our case, we assume fertilizers maintaining the soil quality above the threshold value required by the cash crop for the duration of the growing season. Hence, one can keep making a profit without switching back often to the cover crop. Precisely, in the context of randomized crop switching, fertilizers can manipulate soil quality probability distribution to help increase the θ*. The effective increase in the θ* due to fertilizers is illustrated in figure 4*b*. The minimum amount of soil quality required by the cash crop is the fixed amount *θ*. If the soil quality is less than *θ*, the addition of fertilizers aims to increase it beyond *θ*. However, the actual effect of the fertilizer is perceivable with the complete knowledge of the crop (*θ*), the rate of change of soil quality due to rotation (*a* and *b*) and the effective soil quality distribution.

In this work, we only explore the effect of adding fertilizers on a crop rotation system that is already in a steady state. We model the change in the steady-state soil quality distribution x*, due to fertilizers, as follows:

3.2 $$x_{i}^{*}\to x_{i}^{\prime *}=\frac{x_{i}^{*}+f_{i}}{\sum_{i=0}^{K}(x_{i}^{*}+f_{i})}=\frac{x_{i}^{*}+f_{i}}{1+\sum_{i=0}^{K}f_{i}},$$

where the distribution *f* quantifies the sole effect of fertilizers on the soil quality. For illustration, we choose distribution *f* to be a skewed normal distribution,

3.3 $$f_{i}=\frac{e^{-{\left(i-\mu \right)}^{2}/2\sigma^{2}}\;\text{Erfc}\left[-\frac{\alpha (i-\mu )}{\sqrt{2}\sigma }\right]}{\sqrt{2\pi }\sigma },$$

where *μ* is the mean effect of the fertilizers, *σ* is the corresponding uncertainty measure and *α* represents the skewness in uncertainty towards increased soil quality.

The rates of increase and decrease of the soil quality (*a* and *b*) are intrinsic properties of the crops (cash and cover). We find it might be economically more profitable to change the crop system rather than invest in adding fertilizer to an existing crop system. For example, figure 4*a* shows the disastrous results of adding fertilizers to an *a* = *b* system. Concerning the threshold value, the shaded region shrinks from approximately 31 (without fertilizers) to 20 (with fertilizers). In the right panel for *b* = 1.5 *a* crop system, the addition of fertilizers increases the shaded region from approximately 15 to 19. Here, the addition of fertilizers has helped increase the θ* above the threshold value where profit is possible. To understand the differential results of adding fertilizers, we scrutinize the precise location where the fertilizers take effect—the soil quality. The addition of fertilizers can change the weights associated with the two sums in equation (3.1) in a manner to increase the contribution of the second sum. The second sum in equation (3.1) is associated with the profit, i.e. when the soil quality is higher than the threshold. The (sometimes) increased contribution of the second sum by the addition of fertilizers is discussed in electronic supplementary material (S1.3 Fertilizers). From an economic standpoint, everything else being equal, it might be better to invest in an *a* = *b* system with no fertilizers than an *b* = 1.5 *a* system that would incur the additional cost of fertilizers. Hence the choice of cash–cover crops and an ad hoc assessment of the impact of fertilizers can play an essential role in reaching decisions on fertilizer investment.

## 4. Deterministic sequence

So far, the crop rotation has assumed randomization. Reflecting reality, we analyse patterns of crop rotations such that the order of crops is predetermined. To define a deterministic sequence, we use the following notation, (*α*_1_, *α*_2_, …, *α*_*n*_) where $\alpha_{i}\in Z_{0}^{+}$. For odd *i*, *α*_*i*_ denotes the number of consecutive cover crop, whereas even *i* denotes the number of consecutive cash crop seasons. Thus a sequence of (2, 3, 1, 3) would be a rotation sequence in which the cover crop is planted for two seasons, followed by the cash crop for three seasons and then the cover crop for one followed by the cash crop for three seasons and then the sequence repeats. Thus one cycle of implementing a deterministic sequence takes *α*_1_ + *α*_2_ + · · · + *α*_*n*_ time units. The probability of winning corresponding to a given sequence, *P*_win_ is found from the slope of the average capital trajectory using electronic supplementary material, equation (A.9) by implementing the sequence for long times.

**Figure 5.**
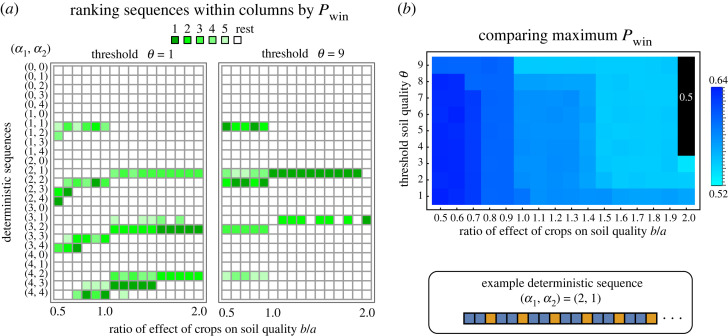
Best deterministic sequences. Deterministic sequences are given by (*α*_1_, *α*_2_) where *α*_1_ is the number of consecutive cover crops followed by *α*_2_ times the cash crops before the sequence repeats, as shown in the inset bottom right. The panels in (*a*) explore the *α*_*i*_ = 1, …, 4 and, for a variety of *b*/*a* ratios (the rate at which the cash crop depletes the soil quality with respect to the rate of replenishment by the cover crop). For select extreme threshold values *θ* = 1 and *θ* = 9, we report the top five sequences *ranked as per highest probability of winning $P_{\mathrm{win}}$ (ranked from dark to light green)*, as long as they have *P*_win_ ≥ 0.5 (with precision set up to two decimal values). As *b*/*a* increases, we see that the best deterministic sequences are not the ones with higher values of *α*_1_ and *α*_2_ but the sequences where the crops switch often. While in (*a*) only *θ* = 1 and 9 are reported, in (*b*) we show only the best *P*_win_ for all combinations of *θ* and *b*/*a* ratios. We see that the benefit coming from the best sequences decreases as the cash crops exploit more of the soil than what the cover crops can replenish (increasing *b*/*a*). The common parameters are *K* = 10, *p* = 0.4, *p*_1_ = 0, *p*_2_ = 0.8.

We scanned all the sequences of type (*α*_1_, *α*_2_) with *α*_*i*_ ∈ [0, 1, …, 4]. Figure 5 shows a Parrondo-like effect for the case of deterministic sequences. If *α*_*i*_ = 0 for any *i*, there is no sequence such that *P*_win_ > 0.5, i.e. growing only one type of crop is a losing strategy. However, for some sequences both *α*_1_ and *α*_2_ are non-zero and we have *P*_win_ > 0.5, i.e. specific combinations of two losing strategies yield a winning one. We observe that the number of such sequences reduces with increasing *θ* and *b*/*a*. We also find that for large *θ*, one needs to switch often between crops to make a profit, especially when *b*/*a* is high. These findings are in agreement with our results from the randomized cropping case.

## 5. Parrondo effect under pathogen pressure

**Figure 6.**
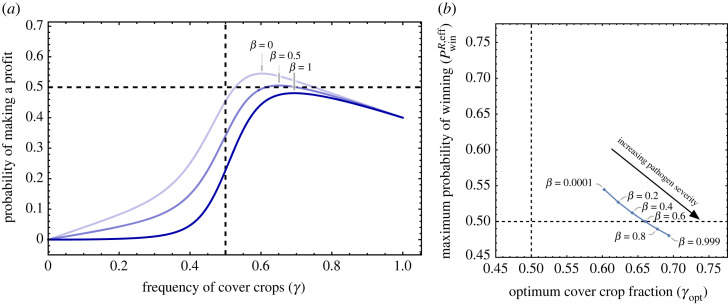
Parrondo under pathogen prevalence. The parameter *β* captures the severity of the pathogen as it affects the cash crop. When *β* = 0 we recover the dynamics as per our simple model as shown in figure 2. In (*a*), we show the probability of winning for different values of *β*. The curves show a maximum for certain values of *γ* that we term as *γ*_opt_ (optimum fraction of cover crops). In (*b*), we show how the position of *γ*_opt_ changes. For an increasing severity of pathogen, we therefore need more cover crops to still make a profit. The parameter values are *K* = 10, *p* = 0.4, *p*_1_ = 0, *p*_2_ = 0.8 along with the maximum pathogen density *L* = 10.

Precision agriculture provides us with means of maximizing profit using exquisitely detailed data. This approach also works for crop rotation sequences [34]. For soils where the quality is not up to the mark, fertilizers have made this factor irrelevant. However, political endeavours promoting cash–cover rotations are still promoted since the benefits of such classical methods can substantially augment precision agriculture. Intercropping is understood to increase crop yield and soil quality [35], but cover crops can also mitigate the impact of pathogens affecting cash crops. How is the profit affected under sustained pathogen pressure? While we have focused on this topic elsewhere in more evolutionary detail [8], here we implement a simpler model to test the validity of the Parrondo effect under pathogen dynamics. We now include a pathogen that affects the cash crop, reducing the yield. The pathogens proliferate on a cash crop but subside when a cover crop is planted. Thus the severity of the pathogen is dynamic and changes along the cropping sequence. The details of the pathogen dynamics are provided in electronic supplementary material, S2 and figure A7. We have captured the severity of the pathogen with the parameter *β*. When *β* is zero, we recover our generic model as per figure 2. Increasing *β*, we see that the Parrondo region shrinks and increases the optimal fraction of cover crops required to recover the best winning probability (figure 6).

Under pathogen dynamics the probabilities of winning for the two crops are affected (*p*, *p*_1/2_). We use the new effective probabilities of winning ($p^{\mathrm{eff}},p_{1/2}^{\mathrm{eff}}$) to compute the effective probability of winning for randomized crop rotation,

5.1 $$P_{\mathrm{win}}^{R,\mathrm{eff}}=q_{1}^{\mathrm{eff}}\sum_{i=0}^{\theta }x_{i}^{*}+q_{2}^{\mathrm{eff}}\sum_{i=\theta +1}^{K}x_{i}^{*},$$

where $q_{1/2}^{\mathrm{eff}}=\gamma p^{\mathrm{eff}}+(1-\gamma )p_{1/2}^{\mathrm{eff}}$. The expressions of the effective probabilities of winning with the different crop types under pathogen pressure are derived in the electronic supplementary material.

## 6. Discussion

Combining two losing games to create a winning scenario seems initially counterintuitive. Parrondo designed this set-up initially intending to explain the dynamics of an imaginary machine that could convert the Brownian motion of particles into work—the Brownian ratchet [36]. The process highlights the positive role of noise in generating ordered structures. Parrondo’s paradox was suggested to have implications in several fields such as economics, biology and social evolution, given the inherent presence of noise in such adaptive systems. Over the past 20 years, this prediction has borne fruit with applications ranging from the classical to the quantum world [37]. Herein, we have introduced Parrondo-like thinking in the field of eco-evolutionary agriculture [8].

Rotating cash and cover crops is a time-tested strategy [38]. The practice of crop rotations is followed worldwide and hence our results can be interpreted in a global context. Indeed, using this theory, one can develop bespoke models for specific systems. The benefit of rotating and diversifying crops has been recently confirmed via a synthesis of long-term studies in maize-based North American cropping systems [39]. The studies show that biodiverse cropping allows for resilience to environmental uncertainty and improves maize yields across different growing conditions, including droughts. In South Asia, the rice–wheat cropping system has been a staple for long; however, new strategies are necessary as the rotation scheme faces a threat from herbicide resistant weeds [40]. From a long-term study from countries in Africa, namely Malawi, Mozambique, Zambia and Zimbabwe, rotations did improve the productivity; however, socio-economical conditions did not provide a profit to the farmers [41]. Therefore, designing a successful rotation schedule is an interdisciplinary challenge and extremely important in yield maximization and sustainability. In the case of randomized cropping, we have leveraged the noise coming from uncertainties in the form of the probabilities of realizing the different outcomes (*p*, *p*_1_, *p*_2_) (see figure 3). While the noise coming from randomized cropping (*γ*) allows for a quantitative analysis, it is the uncertainty in profit generation (*p*, *p*_1/2_) that determines the Parrondo-like effect. In the deterministic setting where sequences are predetermined, we also see the Parrondo-like effect (figure 5). The combination of parameters where the two losing games combine to form a winning game can be extended by fertilizers, but not always. We have shown an example where the addition of fertilizers to the wrong crop system can even decrease the profit margin. We thus highlight the importance of identifying crop–fertilizer combinations before investing in cash crops.

At first glance, our observation of the Parrondo effect in our system is similar to the classical Parrondo’s paradox [12]. However, an important distinction lies between the conventional Parrondo’s effect and the effect that we encounter in our system. In a conventional Parrondo game, the separatrix defining the winning and losing region of the probability space is not a hyperplane (theorem 3.1 of [42]). In our case, it indeed is (see electronic supplementary material, figure A8). In our set-up, the requirement for a cash crop to be a losing strategy is when $p_{1}<\frac{1}{2}$, resulting in the separatrix being a line. Nevertheless, we observe a Parrondo-like effect. Thus, nonlinearity in the games, as seen in the winning probability space, is not a requirement for observing a Parrondo-like effect.

Intending to increase yield, we observe that allowing for mixing games does not lead to a monoculture of the cash crops. The optimal sequences already involve an adequate amount of cover crops so that the soil is not rendered barren. Thus while selecting only for one observable, the cash yield, we have inadvertently also optimized the appropriate soil quality. Furthermore, our model implementation is probabilistic; the cash crop does not always provide a stable return, and the cover crop may not always be a loss-making crop. These uncertainties reflect the unstable nature of the agriculture market economics, weather and climate effects, and the possibilities of crop loss due to disease and pests. Cover crops can replenish the soil, prevent unwanted weed growth and pests, or enhance beneficial insects such as pollinators [26,27]. While we have not explicitly modelled the impact of such specific processes, it would be a future research project to coevolve disease dynamics and winning probabilities. In particular, inclusion of pathogen evolution, as in [8], will complete the eco-evolutionary picture from a Parrondo’s paradox point of view.

We have made several simplifying assumptions to make the complex agricultural scenario amenable for interdisciplinary analysis. This simplification will allow for implementing complicated crop rotation sequences, including seasonal changes or multiple crop types. The methods discussed in this study could form testbeds in small-scale modern agricultural facilities for piloting concepts before being scaled up [43,44]. For example, growing maize and grey-seeded mucuna in summer and winter, followed by cotton and black oats in the following year. Such patterns will extend the game tree, but as long as we can alternate between these sets, a Parrondo-like region will emerge. The Three Sisters approach practised by the indigenous people of the Americas typically plant maize, squash (or pumpkin) and beans together in mounds. The maize provides support for the climbing beans. The beans simultaneously enrich the soil in nitrogen through their association with nitrogen-fixing rhizobacteria, and the squash or pumpkins inhibit the growth of weeds and maintain soil moisture by generating a ground cover [45]. The intercropping technique provides yields that are better than individual monocultures. The mixture of the produce is also nutritionally complementary, providing a wholesome, balanced meal to populations [46]. Including intercropping techniques such as the Three Sisters will add a spatial aspect to Parrondo’s paradox, with multiple games played simultaneously [47]. The games in this case would be proxies for interactions between the different species of crops. Switching between organic and conventional farming has been proposed to be a way to ensure food security [48,49]. Thus the multiple games would experience a fluctuating environment as in Parrondo’s setting. This spatio-temporal cropping technique will be a studied in the classical Parrondo regime and eco-evolutionary agriculture in future work. The calculation of the strict threshold soil quality θ* is a result of us discretizing soil quality in amenable blocks. Further work could account for a smoother threshold function reflecting realistic scenarios and specific crop combinations.

## 7. Conclusion

Fluctuating selection regimes play a crucial role in numerous fundamental and translational biological scenarios. The concept has been discussed before in dynamic cultivation strategies: ‘nomadic’ shifting cultivation and a high growth ‘colonial’ strategy [50]. The nomadic strategy resembles a cover crop game where one makes loss but improves the soil quality and the colonial strategy is akin to the cash crop game where one makes profit if the soil quality is good but that comes at the cost of depleting soil quality and hence, ultimately is a losing game. The study also focuses on resource dynamics by tracking the carrying capacity of each habitat as we do with the soil quality. Our work differs technically as we implement a discrete-time stochastic model, whereas the previous work is a continuous time ODEs-based model. Also, observing a Parrondo-like effect is one of the main results, but not the only result. Firstly, we could predict that it is not always sustainable to use fertilizers, and it is good to know beforehand the system of cash–cover crop before using fertilizers. Secondly, we also study how the pathogen dynamics can decrease the ability to generate profits by a cash–cover crop system. Human interventions in natural processes (either randomly or deterministically determined) can thus be subject to analysis using Parrondo’s paradox. Translational interventions such as adaptive cancer treatment, antibiotic treatment schedules and wildlife conservation techniques to agricultural practices, anthropogenic intervention introduces a dynamic selection regime [51–53]. From designing better antibiotic treatment schedules to lower the probability of resistance evolution to human intervention in the release strategies of gene-drive organisms to control populations, can be instances of Parrondo-like processes. We have thus also included a pathogen component to extend our model in an eco-evolutionary framework. Our study can thus also act as a proof of principle, enhancing the scope and applicability of this approach. While clearly there is much to be done in terms of bringing such concepts closer to the ‘field’ we believe that such translation areas are fertile for applying Parrondo’s paradox. Simultaneously, this thinking provides new opportunities to improve our understanding of Parrondo-like processes in nature.

## Data Availability

Data and relevant code for this research work are stored in GitHub: https://github.com/tecoevo/agriculturalParrondo and have been archived within the Zenodo repository: https://doi.org/10.5281/zenodo.7863147 [54]. The data are provided in electronic supplementary material [55].
